# Haemodynamic changes from prepregnancy to very early pregnancy among women planning to conceive in Southwestern Uganda

**DOI:** 10.1097/HJH.0000000000003988

**Published:** 2025-03-10

**Authors:** Henry M. Lugobe, Carmel M. Mceniery, Musa Kayondo, Janet M. Catov, Joseph Ngonzi, Charles Batte, Bonnie Wandera, Bruce Kirenga, Blair J. Wylie, Adeline A. Boatin, Kwame Adu-Bonsaffoh, David C. Agaba, Ian B. Wilkinson

**Affiliations:** a Department of Obstetrics and Gynecology, Mbarara University of Science and Technology, Mbarara, Uganda; b Division of Experimental medicine, University of Cambridge, Cambridge, UK; c Department of Obstetrics, Gynecology and Reproductive Sciences, School of Medicine, University of Pittsburgh, Magee-Women's Research Institute, Pittsburgh, Pennsylvania, USA; d Lung Institute, Makerere University College of Health Sciences, Kampala, Uganda; e Department of Obstetrics and Gynecology, Columbia University Medical Center, New York USA; f Department of Obstetrics and Gynecology, Massachusetts General Hospital, Harvard Medical School, Boston, Massachusetts, USA; g Department of Obstetrics and Gynecology, University of Ghana medical school, Accra, Ghana; h Department of Physiology, Mbarara University of Science and Technology, Mbarara

**Keywords:** attended blood pressure, haemodynamics, preeclampsia, pregnancy, prepregnancy, sub-Saharan Africa, Uganda, unattended blood pressure

## Abstract

**Introduction:**

Normal pregnancy is associated with cardiovascular changes that enable adaptation to the pregnancy state. We sought to describe the haemodynamic changes from prepregnancy to very early pregnancy in women planning to conceive in southwestern Uganda.

**Methods:**

In this prospective cohort study, we enrolled women in southwestern Uganda planning to conceive. Brachial and central blood pressure, heart rate, cardiac output, stroke volume, and peripheral vascular resistance were assessed prepregnancy and repeated in very early pregnancy.

**Results:**

We studied 86 women with a mean age of 27.8 years (SD ± 4.4). The mean gestational age was 7 (±2) weeks at the time of repeat blood pressure measurement. Brachial systolic and diastolic blood pressure decreased in very early pregnancy (116 ± 11 to 114 ± 8 mmHg and 68 ± 6 to 65 ± 5 mmHg, respectively; *P* < 0.001). Central systolic and diastolic blood pressure also decreased (112 ± 10 to 109 ± 8 mmHg, *P* = 0.003 and 68 ± 6 to 65 ± 5 mmHg, *P* < 0.001, respectively), as did peripheral vascular resistance (1450 ± 581 to 1311 ± 276 dyn/s/cm^5^*P* = 0.038). There was no significant difference in cardiac output (5.3 ± 1.2 vs 5.5 ± 1.1 l/min *P* = 0.146) or stroke volume (64 ± 13 to 66 ± 12 ml, *P* = 0.172).

**Conclusion:**

Significant haemodynamic changes occur in very early pregnancy. Using late first trimester measurements as a baseline for pregnancy induced changes may not be suitable for understanding the full extent of pregnancy induced haemodynamic changes, or provide a reliable substitute for prepregnancy states.

## INTRODUCTION

Normal pregnancy is associated with significant cardiovascular changes that enable adaptation to the pregnancy state. Previous studies have demonstrated a decrease in brachial and central blood pressure (BP), mean arterial pressure (MAP), and peripheral vascular resistance (PVR) in very early pregnancy, while cardiac output (CO) and heart rate (HR) increase [1–4]. Conversely, pregnancies with a decrease in CO and increase in PVR have been associated with preeclampsia and fetal growth restriction [5]. However, most of the studies that have described very early pregnancy changes had a homogenous group of Caucasian women, and whether similar observations occur among women in sub-Saharan Africa is not known [6].

Hypertensive disorders of pregnancy complicate 2–8% of pregnancies [7–9] and remain an important cause of maternal and perinatal morbidity and mortality globally and in sub-Saharan Africa [10,11]. Therefore, the measurement of BP during pregnancy is important; however, standard office measurements may misclassify women with white-coat hypertension. Ambulatory BP is the standard for the diagnosis of hypertension, but this may not be possible in all settings [12]. Unattended office BP measurement (where a device is preset to allow the observer to press the start button and leave the room) is easier to perform and correlates with ambulatory BP more closely than attended office BP reading [13,14]. The difference between attended and unattended blood pressure may be a consequence of white-coat hypertension. However, other studies have shown no difference between attended and unattended office blood pressures [14–16]. None of these studies were conducted among pregnant women, and how these differ in pregnancy is unknown.

Other physiological changes in the cardiovascular system associated with pregnancy include an increase in blood volume by approximately 1.5 l and red cell mass by 10–20% [17,18]. However, the net changes result in haemodilution with a decrease in haemoglobin and haematocrit [17,18]. However, studies in sub-Saharan Africa [19,20] report these changes in pregnancies at advanced gestation when some women have started iron supplementation, and existing studies lack prepregnancy values that would best describe changes occurring as a result of pregnancy.

Therefore, the objective of this study was to describe haemodynamic changes from preconception to very early pregnancy in women planning to conceive in southwestern Uganda.

## METHODS

### Study design and setting

This was a prospective cohort study of women in Southwestern Uganda planning to conceive. This study is nested in a larger study aiming to establish the feasibility of recruiting a prospective cohort of women prior to conception and following them through the postpartum period, with cardiovascular and metabolic measurements obtained prepregnancy and at defined intervals throughout the follow-up period. This study was conducted at Mbarara Regional Referral Hospital (MRRH), between January 2023 and June 2024. The MRRH is a government-funded public hospital that conducts approximately 9000 deliveries annually. The hospital has both outpatient and inpatient maternity care facilities. Prospective study participants were recruited through the family planning clinic at the MRRH, radio advertisements, word of mouth, church announcements, and display of posters at markets and churches. The majority of participants were recruited from the family planning clinic as the study team reached out to women who were discontinuing family planning because they were planning a pregnancy.

### Participants

We included all women of reproductive age planning to conceive. We excluded participants who had received assisted reproductive technology. Pregnancy testing was performed using a urine HCG strip at enrollment. Participants were then provided urine HCG strips to perform pregnancy tests every menstrual cycle while at home until they had a positive test or for up to 6 months. All women were given folic acid tablets (5 mg) to take daily during the study period.

### Data collection and study variables

Sociodemographic (age, marital status, level of education, smoking, alcohol intake, weight, and height), medical (HIV serostatus, known history of hypertension and diabetes mellitus), and obstetric factors (parity, previous history of hypertension in pregnancy) were captured through participant interviews at enrolment. Body mass index (BMI) was calculated as the weight in kilograms divided by the height in meters squared. BMI was classified as underweight (<18.5 kg/m^2^), normal weight (18.5–24.9 kg/m^2^), overweight (25–29 kg/m^2^) and obese (≥ 30 kg/m^2^) at data analysis.

Haemodynamic assessments were performed on two occasions: prior to pregnancy and very early pregnancy between 6 and 12 weeks of gestation by the last menstrual period. Assessments were performed after 10 min of seated rest. The participant was seated on a chair with her back supported, feet on the floor, arm supported, and cubital fossa at the level of the heart. Brachial BP and HR were measured on the left arm using an automated oscillometric BP measuring device (Omron HEM-907; Omron Healthcare Co. Ltd, Japan), which has been validated for use in pregnancy [21]. Standard cuff sizes were used according to the participants’ arm circumference. Blood pressure was measured initially with the clinician in the room (attended BP). Unattended BP was then measured using the automated setting on the BP device while the clinician was not in the room, as previously described by D'sa *et al.*[22]. Brachial BP measurements were then repeated using a second oscillometric method, a Vicorder (Skidmore Medical Ltd, UK), which is a cuff-based device that has been used before in pregnancy [23,24]. Brachial pressure waveforms were then recorded to yield noninvasive estimates of central BP, heart rate, cardiac output, stroke volume, mean arterial pressure (MAP), augmentation pressure, and augmentation index using a proprietary algorithm [24]. Peripheral vascular resistance was calculated as PVR (dynes s^−1^ cm^−5^) = MAP (mmHg) × 80/CO (l/min). All haemodynamic assessments were performed in triplicate and the mean value of the second and third readings were used.

Blood was collected for assessment of full blood count (Beckman Coulter ACT 5 Diff CP haematology analyzer) at the prepregnancy and 6–12 weeks of pregnancy visit.

### Data analysis

Study data were collected and managed using Research Electronic Data Capture (REDCap) tools hosted at Mbarara University of Science and Technology [25–27]. Statistical analysis was performed using Stata version 17 (Statacorp, College Station, TX, USA). Continuous data were presented as means and standard deviations, while categorical variables were presented as frequencies and percentages. Prepregnancy and early pregnancy BP measurements and hemodynamic and biochemical data were compared using two-tailed paired t-tests.

### Ethical consideration

This study was approved by the Mbarara University of Science and Technology Research Ethics Committee (MUST-2022–491), Mbarara Regional Referral Hospital Administration, and Uganda National Council for Science and Technology (UNCST- HS2452ES). All participants provided written informed consent to participate in the study.

## RESULTS

There were 86 women that conceived and attended the early pregnancy visit during the study period. At the pregnancy visit, the mean (standard deviation) gestational age was 7 (±2) weeks. The baseline participant characteristics are presented in Table 1.

**TABLE 1:** Showing prepregnancy participant characteristics

Characteristic *n* = 86	Categories	Mean (SD)
Age		27.8 (4.4)
Body mass index (kg/m^2^)		26.2 (±4.7)
Characteristic *N* = 86	Categories	*N* (%)
Marital status	Married	86 (100)
Level of education	None	3 (3.5)
	Primary and below	14 (16.3)
	Secondary and tertiary	69 (80.2)
HIV status	Negative	77 (89.5)
	Positive	9 (10.5)
Known history of Hypertension	No	83 (96.5)
	Yes	3 (3.5)
Known history of DM	No	86 (100)
Parity	Nulliparous	6 (7.0)
	1–4	79 (91.8)
	≥5	1 (1.2)
History of pregnancy hypertension	No	80 (93.0)
	Yes	6 (7.0)
History of smoking	No	86 (100)
History of alcohol intake	No	81 (94.2)
	Yes	5 (5.8)
Body mass index (kg/m^2^)	<30	69 (80.2)
	≥30	17 (19.8)

DM, diabetes mellitus; SD, standard deviation.

Systolic and diastolic pressure BP, mean arterial pressure (MAP), and peripheral vascular resistance all decreased in early pregnancy, while heart rate increased, as anticipated. There was a small increase in cardiac output and stroke volume, but these changes were not statistically significant between prepregnancy and early pregnancy as shown in Table 2.

**TABLE 2:** Haemodynamic characteristics of women prepregnancy and early pregnancy

Characteristic *n* = 86	Prepregnancy Mean (SD)	Early pregnancy Mean (SD)	*P* value
Clinic BP			
Systolic BP (mmHg)	116 (10)	112 (8)	<0.001
Diastolic BP (mmHg)	76 (8)	70 (6)	<0.001
Heart rate (bpm)	78 (9)	83 (10)	<0.001
Haemodynamics *n* = 85			
Brachial SBP (mmHg)	116 (11)	114 (8)	0.019
Brachial DBP (mmHg)	68 (6)	65 (5)	<0.001
Mean arterial pressure (mmHg)	89 (8)	87 (6)	<0.001
Heart rate (bpm)	85 (14)	85 (10)	0.772
Stroke volume (ml)	63.7 (13.1)	65.8 (12.4)	0.172
Cardiac output (l/min)	5.3 (1.2)	5.5 (1.1)	0.146
Peripheral vascular Resistance (dyn s^−1^ cm^−5^)	1450.2 (580.7)	1311.3 (275.7)	0.038
Aortic systolic BP (mmHg)	112 (10)	109 (8)	0.003
Aortic diastolic BP (mmHg)	68 (6)	65 (5)	<0.001
Augmentation pressure (mmHg)	7.7 (5)	7.2 (4)	0.349
Augmentation index (%)	16.5 (8.6)	15.5 (7.3)	0.285

BP, blood pressure; SD, standard deviation.

There was a significant decrease in haemoglobin, haematocrit, red blood cell counts, mean corpuscular volume and mean corpuscular haemoglobin in early pregnancy. There were no significant differences in the white blood cell or platelet counts as shown in Table 3.

**TABLE 3:** Full blood count of women prepregnancy and early pregnancy

Characteristic *n* = 86	Prepregnancy Mean (SD)	Early pregnancy Mean (SD)	*P* value
White blood cells (10^3^/μl)	8.0 (2.9)	8.1 (3.1)	0.766
Haemoglobin (g/dl)	13.1 (1.8)	12.1 (1.8)	<0.001
Haematocrit (%)	40.1 (4.5)	36.9 (4.3)	<0.001
Red blood cells (10^6^/μl)	4.7 (0.4)	4.5 (0.5)	0.0003
Mean corpuscular volume (fl)	85.8 (8.3)	80.8 (12.8)	0.0001
Mean corpuscular haemoglobin (pg)	28.6 (5.7)	27.2 (2.9)	0.0242
Mean corpuscular haemoglobin concentration (g/dl)	32.7 (1.6)	33.2 (2.2)	0.0753
Platelets (10^3/μl)	223.8 (63.4)	224.7 (63.3)	0.8845

SD, standard deviation.

Unattended systolic BP was lower than attended systolic BP, whereas the unattended heart rate was higher than the attended heart rate. However, there was no difference in diastolic BP between attended and unattended readings, as shown in Table 4.

**TABLE 4:** Attended and unattended blood pressure and heart rate measurements

Characteristic	variable	Attended mean (SD)	Unattended mean (SD)	Mean difference (95% CI)	*P* value
Prepregnancy	Systolic BP	116.3 (11.6)	114 (10.7)	2.3 (1.6,3.0)	<0.0001
	Diastolic BP	76 (9.7)	76.5 (9.7)	−0.5 (-1.1,0.1)	0.0981
	Heart rate	78.9 (10)	80.1 (10)	−1.2 (−1.8,-0.6)	<0.0001
Early pregnancy	Systolic BP	112.4 (8.1)	111.1 (8.4)	1.3 (0.3,2.3)	0.0115
	Diastolic BP	70.3 (6.2)	71.1 (7.1)	−0.8 (−1.8,0.3)	0.1418
	Heart rate	83.4 (10)	84.3 (9.1)	−0.9 (−1.7,-0.03)	0.0424

CI, confidence interval; SD, standard deviation.

## DISCUSSION

In early pregnancy, systolic blood pressure, diastolic blood pressure, mean arterial pressure, and peripheral vascular resistance decreased, while the heart rate increased. However, the increases in cardiac output and stroke volume were not statistically significant. There was a decrease in haemoglobin, haematocrit, and red blood cell counts in early pregnancy. The unattended systolic blood pressure was lower than the attended systolic blood pressure, while the unattended heart rate was higher than the attended value. However, there was no difference in the diastolic blood pressure.

The changes in blood pressure values and peripheral vascular resistance were similar to the observed changes in other studies outside Africa [1–4]. Although we did not observe any significant changes in cardiac output and stroke volume, this was again similar to previous findings [3]. The decrease in blood pressure is likely due to the observed decrease in vascular resistance, which itself could result from smooth muscle relaxation in response to progesterone, nitric oxide, prostaglandins, or atrial natriuretic peptide [17], although we did not measure any of these substances in the current study.

The observed decrease in haemoglobin and haematocrit levels is likely due to a pregnancy-associated increase in plasma volume, which is disproportionate to the increase in red blood cell volume. Indeed, pregnancy-induced haemodilution with a reduction in haematocrit has been well described [17].

There was a significant difference between attended and unattended systolic blood pressures. However, we observed no such difference between attended and unattended diastolic blood pressures. Some previous studies showed no difference in attended and unattended office blood pressure, although attended blood pressure tended to be higher than unattended blood pressure [14–16]. These studies included adult participants, and the women were nonpregnant.

The strength of this study is that, to our knowledge, it is the first study in sub-Saharan Africa to include prospective longitudinal data on maternal cardiovascular haemodynamics from preconception to very early gestation. However, our study has some limitations. This was a prospective study conducted at a single site with a small sample size and may not be generalizable across sub-Saharan Africa. We also used a noninvasive technique to measure cardiac output and stroke volume, which may have greater variability compared with invasive techniques or those based on Fick's principles. Nevertheless, our data are comparable with previous findings based on an inert gas rebreathing technique.

## CONCLUSION

Significant haemodynamic changes occur in very early pregnancy. During clinical care, using late first-trimester measurements as a baseline may not be the best measure to understand haemodynamic changes occurring during pregnancy. The ability to demonstrate detailed haemodynamic changes from prepregnancy is important to pave the way for further longitudinal research in cardiovascular changes in pregnancies in sub-Saharan Africa, where complications such as preeclampsia are associated with severe maternal morbidity and mortality.

## ACKNOWLEDGEMENTS

The authors would like to thank the study participants for their participation. Special thanks also go to the research assistants Dr Chrispus Mumbere, Patience Naiga, Florida Tusiimiraho, Stewart Agaba, Gerald Mwebesa, Molly Busingye, Yona Mbalibulha, Alan Babweteera, Jonasi Agaba, and Rachel Akampwera for their efforts during this study. We are grateful to the administration of the MRRH for their support during the execution of this study.

Funding: Research reported in this publication was supported by the Fogarty International Center of the National Institutes of Health under award number D43TW011401 to HML. The content is solely the responsibility of the authors and does not necessarily represent the official views of the NIH. The research reported here was supported by the Commonwealth Scholarship Commission and the Foreign, Commonwealth and Development Office in the UK to HML. The author is grateful for their support. All views expressed here are those of the author(s) not the funding body. HML is funded by the Cambridge Trust. This research was supported, in part, by the NIHR Cambridge Biomedical Research Centre (BRC-1215-20014) to CMM. The views expressed are those of the authors and not necessarily those of the NIHR or the Department of Health and Social Care. The funders had no role in study design, data collection, and analysis, decision to publish or preparation of the manuscript.

### Conflicts of interest

There are no conflicts of interest.
